# Comparison of knowledge on stroke for stroke patients and the general population in Burkina Faso: a cross-sectional study

**DOI:** 10.3934/publichealth.2020056

**Published:** 2020-09-21

**Authors:** Christy Pu, Jiun-Yu Guo, Yu-Hua-Yeh, Placide Sankara

**Affiliations:** 1 Institute of Public Health, National Yang-Ming University, Taipei, Taiwan; 2 Department of Medicine, Taipei Veterans General Hospital, Taipei, Taiwan; 3 Ministry of Health, Burkina Faso

**Keywords:** preventive behavior, stroke patients, knowledge, Burkina Faso

## Abstract

**Background:**

In many parts of Africa, there is limited information on awareness of symptoms of stroke, risk factors for stroke and willingness for stroke prevention, both in the general population and in people with stroke. Knowledge and preventive efforts for stroke in patients with a history of the illness are rarely investigated. This study aims to investigate awareness of stroke symptoms in stroke patients who were admitted to hospitals within 72 hours of a confirmed stroke event in Burkina Faso. This study also aims to investigate preventive behavior for stroke for the general population.

**Methods:**

Face-to-face interviews were conducted with the participants. The sample included 110 first-time stroke patients who had been admitted to one of three tertiary teaching hospitals in Burkina Faso within 72 hours and 750 participants from the general population, who were recruited through clustered sampling. Knowledge of stroke warning signs and current and future efforts on stroke prevention were also assessed.

**Results:**

Only 30.9% of the stroke patients believed that they were at risk before the stroke episode. Obvious warning signs were unfamiliar to both groups. Only 1.3% of the respondents from the general population group knew sudden weakness face arm or leg as a sign of stroke. For all future efforts in stroke prevention, stroke patients demonstrated significantly lower willingness to undertake behavioral changes than the general population. Sixty-six percent and 85% of the stroke patients and the general population, respectively, were willing to take steps to reduce blood pressure.

**Conclusion:**

Public education on stroke warning signs and strategies to increase willingness to engage in preventive behaviors are urgent in African countries. Strategies to improve public awareness for developing countries such as Burkina Faso should be designed differently from that of developed countries to incorporate local beliefs.

## 1. Background

Studies have demonstrated that cardiovascular diseases are the second most common cause of death in Africa, and stroke was the fourth leading cause of all deaths in 2015 [1]. Despite several public health initiatives to prevent and control stroke and other noncommunicable diseases (NCDs) in Africa [2], effective stroke treatment requires patients to be willing to initiate preventive measures. In addition, being aware of the symptoms and signs of stroke, as decreasing the time between stroke onset and arrival at the first treating hospital is vital for stoke patients [3]. This belief is in line with the “time is brain” concept, which states that the passage of time has an deleterious effect on ischemic brain tissue [4] and the Stroke Heroes Act FAST (face, arm, speech, time) campaign [5].

There are studies on the awareness of symptoms signs for stroke [6],[7]; however, studies on this topic have rarely been conducted for stroke patients. Investigating awareness of symptom signs for stroke patients and compare this level of awareness to the general population is essential because such comparison provides information on whether people who are at high risks of stroke do in fact have higher awareness. Such comparative studies remain rare, even for developed countries. People with a history of stroke represent the group with the highest stroke risk, and studies have shown that recurrent stroke was not uncommon among stroke patients and could be fatal [8],[9].

Most studies on stroke symptom awareness have suggested that awareness was generally low in the general population, and such awareness varied by demographic and socioeconomic factors, such as age and education [10]–[12]. Pancioli et al. used a sample of 2,642 individuals in Ohio, United States (US), to reveal that only 57% of the respondents correctly listed at least one stroke warning signs [12]. A study conducted in the US suggested that Black women had twice the incident stroke risk of White women [13]. However, awareness of stroke symptoms did not vary by race and ethnicity [14]. Most of the studies on stroke warning signs have been conducted in high-income developed countries. Of the few studies conducted in Africa, Murray et al. indicated that in Ghana, awareness of stroke symptoms was extremely low and that one-sided weakness was the only symptom identified [15]. A hospital-based study conducted in Nigeria revealed that of the 225 patients diagnosed as having hypertension, diabetes, or both, only 39.6% could identify at least one stroke warning sign. Ayanniyi et al. demonstrated that the baseline knowledge regarding stroke was poor in Nigeria [16]. More evidence from African countries is vital as these countries are undergoing a transition from communicable diseases to NCDs as the main causes of death and disability.

Other than limited studies from African countries, another gap in this branch of research is yet to be fulfilled; that is, the comparison of stroke awareness of the general population to people who have experienced stroke. Such comparison provides additional information on whether people who are at high risks of stroke do in fact have higher awareness. Such comparative studies remain rare, even for developed countries. People with a history of stroke represent the group with the highest stroke risk, and studies have shown that recurrent stroke was not uncommon among stroke patients and could be fatal [8],[9].

Burkina Faso is a landlocked francophone country situated in sub-Saharan Africa. The land size is about 272,967.47 square kilometers, with an estimated 18,450,494 inhabitants. Burkina Faso is faced with the burden of NCDs; stroke is the fourth leading cause of death after influenza and pneumonia, malaria, and diarrheal diseases. In 2016, there was 7985 new incidences of stroke in Burkina Faso. Despite it is lower compared with other African countries, however, it represented one of the largest increase in percentage change in age-standardized rates during the period 1990–2016. The overall percentage change in age-standardized rates during the period 1990–2016 was 9.8% in the country, while the overall rate in Western sub-Saharan Africa was −5.7% [17]. Stroke deaths in the country reached 9,118 or 6.29% of the annual total death [18]. These statistics underscore the urgency with which the phenomenon of stroke needs to be studied in Burkina Faso. Indeed, the WHO STEPS Country Reports [19] indicate that the prevalence of tobacco consumption was high (19%) and that 13.4% and 4.5% of the population were overweight and obese, respectively. In addition, 17.6% had high blood pressure, 4.9% had diabetes, and 3.5% had hypercholesterolemia. These include some of the main risk factors for stroke. However, the country lacks data regarding stroke awareness in the population.

This study aims to investigate awareness of stroke symptoms in stroke patients who were admitted to hospitals within 72 hours of a confirmed stroke event in Burkina Faso. This study also aims to investigate preventive behavior for stroke for the general population.

## 2. Methods

### 2.1. Sampling method

#### 2.1.1. General population: First stage: urban-rural study site selection process (Stratified selection of the primary sampling units)

We first stratified the country into 13 strata (strata are defined by the 13 administrative regions). Then, using a simple random selection process, we selected four primary sampling units (PSUs). From the 13 strata, we randomly selected two strata (using ballot method). Then, we listed all the provinces from which each of the selected strata were derived and randomly selected one province. Usually, each province had only one urban commune; however, where there was more than one urban commune, one was randomly selected. Finally, the rural communes of each selected province was listed, and then one was randomly selected from each province.

At the provincial level, Kossi and Comoé were selected from each of the two selected regions. At the urban commune level, urban Nouna (PSU) was the only urban commune of Kossi province; urban Banfora (PSU) was randomly selected from the two urban communes that constitute Comoé province. At the rural commune level, Doumbala (PSU) and Bérégadougou (PSU) were randomly selected. These areas were the target sources of the respondents.

#### 2.1.2. Second stage: household selection process (Systematic selection of households)

The sampling frame included all households in these areas. We conducted an exhaustive enumeration of all households in each PSU. Then, we randomly selected the households to be surveyed using random digits.

#### 2.1.3. Third stage: respondent selection process (Random selection using Kish method)

Within a selected household, we randomly selected an individual aged at least 20 years old by using the Kish method [20]. If the selected person was not available, a later appointment was made for the interview. If the selected person was unwilling to participate, no substitution was made in that household, and we considered the next household.

### 2.2. Sample size estimation

Based on the 2006 national statistics, 20,778 and 13,594 households were identified in urban Banfora and urban Nouna, respectively; 5044 and 2079 households were identified in rural Doumbala and rural Bérégadougou, respectively. The population size in the urban areas included the total number of households in each urban area (20,778 + 13,594 = 34,372), which provided a minimum sample size of 380 households. The same procedure was used to determine the sample size in the rural areas, which provided a minimum sample size of 365 households. The final allocation of sample size of a selected area was proportional to the number of households in the area. Therefore, the final sample included 230 participants from urban Banfora, 150 from urban Nouna, 258 from rural Doumbala participants (rounded up to 260), and 107 from Bérégadougou (rounded up to 110).

The total number of participants from the general population was 750. The sampling method allowed us to screen urban and rural populations from diverse ethnic, cultural, and educational backgrounds. We employed the following inclusion criteria: All selected individuals had to be constant residents of these areas for at least 1 year, aged at least 20 years, and able to provide relevant information. The exclusion criteria were unwillingness to participate and being a healthcare professional.

### 2.3. Patients with a diagnosis of stroke or transient ischemic attack

Multicenter, prospective, and consecutive study methods were used in this study. We conducted the survey within three tertiary teaching hospitals: the university hospitals of Yalgado Ouédraogo, Blaise Compaoré, and Souro Sanou. These were referral hospitals that offered specialized neurological care, and they were selected according to their service coverage and their capacity for treating strokes or transient ischemic attack (TIA). They were the only centers that provided treatment for stroke or TIA patients in the country.

In each selected hospital, at the emergency department (ED) and neurology units, we consecutively recruited and interviewed patients with a diagnosis of stroke or TIA. Typically, all patients entered the hospital at the ED. A doctor evaluated and confirmed their stroke or TIA cases through clinical examination of patients presenting with neurological symptoms. A neurologist verified the stroke diagnosis, and a brain computed tomography (CT) scan was performed. For the purpose of this study, only neurologist diagnoses were considered as the gold standard. Patients with a final stroke or TIA diagnosis confirmed by CT or MRI were analyzed. Therefore, those with nonstroke neurological deficits were excluded.

Study physicians (investigators) explained and provided detailed information regarding the study procedures to all eligible patients or their legal guardians. We only interviewed those who consented to participate and signed the informed consent form. To minimize in-hospital stroke education post stroke, all patients were interviewed within 72 hours after admission.

Before interviewing the patients, we assessed their stroke severity with the National Institutes of Health Stroke Scale [21] and assessed their mental state with the Mini-Mental State Examination test [22]. We excluded patients unable to communicate (comatose patients and patients with speech difficulties), those with subarachnoid hemorrhage, patients presenting an impaired mental status (such as unconsciousness or disorientation), and patients whose relatives were not available to provide information. The sample size was calculated using the formula (1):

$$\text{N}=\frac{{\text{Z}}^{2}\text{xP}(1-\text{P})}{{\text{e}}^{2}}$$ (1)

where Z = α error (5%); P = proportion of people with an overall cardiovascular risk factor of at least 30% in previous findings in the country (7.8%); e = precision (5%). We thus estimated the sample size as 110 patients.

### 2.4. Data collection

We collected the data in 4 months through direct interviews from June 1 to September 30, 2017. The interviews were face-to-face and utilized a modified standardized questionnaire. Field teams with previous experience were trained by the project leader to conduct the survey. All the interviewers responsible for data collection met the required ethical standard. Informed consent was obtained before interviews for all individual participants. Quality-control checks were adopted when a questionnaire was returned from the interviewer. A pilot test was conducted before official data collection. Interviews were performed in participants' homes or at the hospital (or other suitable locations) and at times that were suitable, convenient, and that could ensure participants' confidentiality.

### 2.5. Study instruments

To suit the local sociocultural contexts and practices, the perception and practical aspects of the questionnaire were constructed and modified from previous studies. These studies already assessed perceptions and attitudes toward stroke in sub-Saharan Africa [16],[23],[24]: Participants either completed the survey on their own or provided verbal responses to items read by an investigator. The survey lasted approximately 30 min.

For stroke warning signs, we categorized warning signs into three categories. Stroke may have different presentations for different patients. For example, certain presentations of stroke may include vision loss; however, not all patients may experience this symptom. The first category (Category A) involved obvious symptoms, including (1) sudden loss of balance or coordination; (2) sudden numbness of the face, arm, or leg; (3) sudden weakness of the face, arm, or leg; (4) sudden trouble speaking or understanding speech; (5) sudden severe headache with no known cause; (6) sudden difficulty walking; (7) tremor; (8) sudden dizziness and (9) sudden confusion. These symptoms are in line with the stroke symptom guidelines [5]. The second category (Category B) involved those with known stroke warnings signs that may not be as typical. These warning signs include (1) sudden respiratory disturbances; (2) nausea or vomiting; (3) sudden difficulty seeing in one or both eyes. The final category included “wrong answers” or answers not typical for stroke warning signs (Category C). This category was included to avoid the respondents simply checking all items for convenience. Warning signs included in this categories were (1) sudden fear or anxiety; (2) sudden memory loss; (3) chest pain or chest tightness;(4) diarrhea and (5) fever and sweating.

We then asked the respondents to report whether they had implemented stroke preventive actions before the survey, and if not, would they initiate behavioral change to prevent stroke (or recurrent stroke for the stroke patient group) in the near future. These actions were listed out in the questionnaire and included the following: (1) attempting to control blood pressure; (2) attempting glycemic control; (3) following a low cholesterol diet; (4) exercising; (5) quitting cigarette smoking; (6) quitting alcohol drinking; (7) stopping oral contraceptives and (8) reducing stress. Validity of stroke warning signs were assessed by two neurologists, and the complete questionnaire was sent to five public health experts for revision.

## 3. Results

Table 1 displays the sample characteristics for stroke patients (*n* = 110) and the general population (*n* = 750). The stroke patient group was much older, with 30% of the respondents being more than 65 years old, whereas the corresponding figure for the general population was 4%. Life expectancy for Burkina Faso was only 60 and 61 for men and women, respectively in 2016 [25]. Furthermore, stroke is more frequent among the elderly people, which may explain the difference in age distribution. No significant difference in gender was observed between the two groups. Similarly, no significant difference in education level and religion was noted for the two groups. A higher proportion of the stroke patient group reported having hypertension (72%). However, a much lower proportion of the stroke patient group reported having diabetes (39.1% in the stroke patient group and 87.9% in the general population group). A higher proportion of the general population reported a family history of stroke. In the stroke patient group, 38.2% were current smokers, whereas the corresponding number was 28.9% for the general population group. Unsurprisingly, a much higher proportion of the stroke patients reported having fair and poor health (as evaluated before the stroke event) compared with the general population (*p* < 0.001).

**Table 1.** Sample characteristics.

	Total		Stroke patient		General population		p-value
	n = 860		n = 110		n = 750	
Age	n	%	n	%	n	%	
≤65	779	90.6	59	53.64	720	96	<0.001
>65	81	9.4	51	46.36	30	4	
Sex (male)	665	77.3	78	70.91	587	78.27	0.085
Education							
No education	475	55.2	59	53.64	416	55.47	0.85
Primary	177	20.6	22	20	155	20.67	
Secondary and above	208	24.2	29	26.36	179	23.87	
Religion							
Catholic	171	19.9	24	21.82	147	19.6	0.097
Muslim	438	50.9	56	50.91	382	50.93	
Protestant	182	21.2	16	14.55	166	22.13	
Traditional and others	69	8.0	14	12.73	55	7.33	
Occupation							
Housewives	116	13.5	21	19.09	95	12.67	<0.001
Employed	48	5.6	13	11.82	35	4.67	
Unemployed	43	5.0	14	12.73	29	3.87	
Self-Employed	546	63.5	42	38.18	504	67.2	
Retired/Others	107	12.4	20	18.18	87	11.6	
Disease history							
Hypertension (Yes)	128	14.9	80	72.7	48	6.4	<0.001
Diabetes (Yes)	702	81.6	43	39.1	659	87.9	<0.001
Family history of stroke (Yes)	715	83.1	23	20.9	692	92.3	<0.001
Current smoker (Yes)	259	30.1	42	38.2	217	28.9	0.048
Regular exercise (Yes)	190	22.1	25	22.7	165	22.0	0.864
Self-rate health(before stroke for stroke patients)							
Excellent	171	19.9	12	10.91	159	21.2	<0.001
Good	405	47.1	40	36.36	365	48.67	
Fair	219	25.5	46	41.82	173	23.07	
Poor	65	7.6	12	10.91	53	7.07	

**Figure 1.**
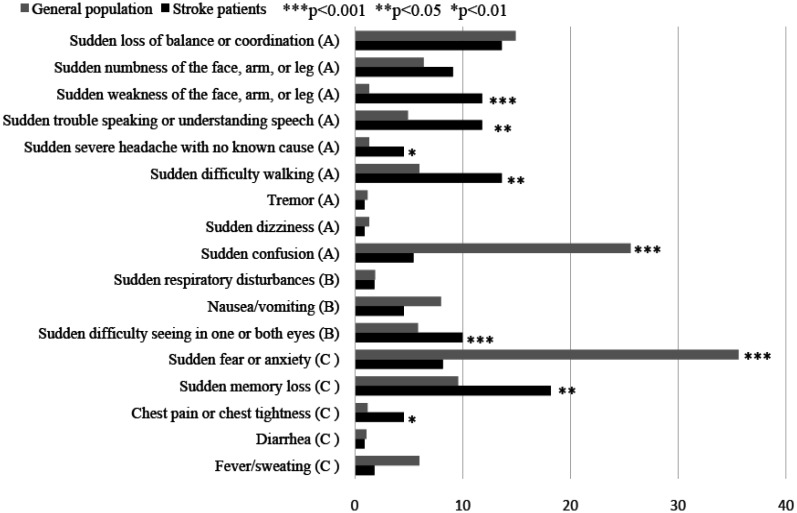
Awareness of warning sign for stroke (% responded “yes” for each question).

Awareness of stroke warning signs according to stroke status is presented in Figure 1. The number of participants who reported “yes” for Category A was surprisingly low. Only approximately 15% of the respondent from both groups believed that sudden loss of balance or coordination was a stroke symptom. Only 1.3% of the respondents from the general population group knew that sudden weakness of the face, arm, or leg was a symptom of stroke. Less than 1% of respondents in both groups knew that tremors and sudden dizziness could be stroke warning signs. The proportion of respondents who could accurately state the correct warning sign for this category remained low, even for stroke patients. For warning signs in Category B, the two groups displayed no significant differences in answers. However, for all correct warning signs, the percentage of those who indicated correctly remained low. Category C was an interesting category as it was comprised warning signs that were not warning signs for stroke. More than 35% of the respondents in the general population group believed that sudden fear or anxiety was a warning sign for stroke, whereas only approximately 8% of former stroke patients believed that it was a warning sign for stroke. A significantly higher proportion of stroke patients believed that sudden memory loss was a warning sign for stroke. Similarly, a significantly higher percentage of respondents from the stroke group believed that chest pain or chest tightness was a warning sign.

Almost 15% of the respondents from both groups believed that stroke was caused by supernatural causes (Table 2). Only 30.9% of the stroke patients believed they were at risk of stroke before the stroke episode. A higher proportion was even observed for the general population group (48.4%). Over 90% in both groups reported not having any stroke preventive behaviors.

For all future efforts in stroke prevention, stroke patients performed significantly worse than the general population. Other than quitting smoking, attempting to control blood pressure and glycemic index were the most popular prevention methods reported by both groups. Of those who indicated they were current smokers, only 33.3% of the stroke patients reported they would quit smoking in the future. A much higher percentage was observed for the general population group (86.8%).

Figure 1 shows Awareness of warning sign for stroke, stratified by general population and stroke patients who were admitted to the emergency room within 72 hours. Stroke warning signs were categorized as “obvious warning sings” (Category A), “stroke warning signs that may not be as typical” (Category B), and “wrong warning signs” for stroke (Category C).

**Table 2.** Stroke perception and preventive practices.

	Total (n = 860)	%	Stroke patients (n = 110)	%	General population (n = 750)	%	p-value
Believed stroke is caused by supernatural causes	131	15.23	23	20.91	108	14.4	0.182
Do you believe that you are at risk of stroke?^1^							
Yes	397	46.16	34	30.91	363	48.4	<0.001
No	172	20	73	66.36	99	13.2	
Don't know	291	33.84	3	2.73	288	38.4	
Stroke prevention (no, not any)^2^	778	90.47	84	76.36	694	92.53	<0.001
Of those who said no, will do any prevention from now? (Yes)							
Attempting to control blood pressure	708	82.33	73	66.36	635	84.67	<0.001
Attempting glycemic control	599	69.65	43	39.09	556	74.13	<0.001
Following a low cholesterol diet	42	4.88	1	0.91	41	5.47	0.038
Doing exercise	4	0.47	1	0.91	3	0.4	0.464
Quit cigarette smoking^3^	201	77.61^4^	14	33.33^4^	187	86.18^4^	<0.001
Quit alcohol drinking	242	28.14	10	9.09	232	30.93	<0.000
Stop oral contraceptives	343	39.88	18	16.36	325	43.33	<0.000
Reduce stress level	23	2.67	4	3.64	19	2.53	0.503

Note: ^1^ For stroke patients, this question refers to whether the respondent believed he or she was at risk before the stoke event. ^2^ Including (1) attempting to control blood pressure; (2) attempting glycemic control; (3) following a low cholesterol diet; and (4) exercising; (5) quitting cigarette smoking; (6) quitting alcohol drinking; (7) stopping oral contraceptives; and (8) reducing stress. ^3^ For respondents who reported they were “current smokers” only. ^4^ Percentage represents those who were current smokers.

## 4. Discussion

Stroke is the major cause of disability in low and middle income countries, and its incidence has doubled during the past four decades [26]. Delay in hospitalization is one of the main factors that contribute to high mortality and morbidity in stroke [27]. This was the first study that investigated awareness of stroke warning signs in Burkina Faso, where stroke is a major contributor to disability. We discovered that awareness of stroke warning signs was low in both the stroke patient and general population groups. In our study, we categorized stroke warnings signs into typical signs, nontypical signs, and inaccurate signs. Inaccurately identifying a wrong warning sign is of less concern than failing to identify a correct warning sign. The former may initiate unnecessary medical treatment but is less likely to lead to irreversible fatal outcomes, as delayed treatment on the other hand would.

This study has several notable merits and limitations. First, in addition to collecting data from the general population, we also collected data from stroke patients. This approach provided a range of essential information. First, since stroke patients represented people at the highest risk of stroke (given that they had a history of stroke), we were able to determine whether people at high risks had sufficient awareness relative to people who were at lower risk. Second, by having a comparison group (general population versus stroke patients), we could determine whether certain responses were high or low in a relative, rather than simply absolute, sense. In addition, the percentage of respondents who identified an accurate warning sign from either group could be cross checked with responses from the other group. For example, any systematic trend could be identified. The limitations of this study should also be noted. First, while the general population sample was representative of the target population, the stroke patient sample was less representative, as it was collected from selected hospitals. In addition, we only included stroke patients who were able to communicate, thus there may be a selection bias towards patients with milder strokes. However, data from stroke patients are rare, and we included three tertiary hospitals, and this was much more representative than other studies that involved only a single hospital. Recall bias was another limitation of the study. Recall bias is usually a limitation of survey studies, and this study could not eliminate it.

Timely treatment of hypertension and appropriate lifestyle changes may help decrease the incidence of stroke. Without public knowledge and the right perceptions and practices, stroke burden cannot be reduced. Therapy should start immediately upon stroke onset; furthermore, the effect of treatment reduces significantly if given later than 6 h after stroke onset [28]. The percentage of respondents who accurately identified stroke warning sign was significantly lower than those in developed countries. For example, in a study conducted in the US, up to 85% of the respondents selected three or more (out of a total of four) correct stroke warning signs [29]. Another study on women, also conducted in the US, discovered that 51% identified sudden weakness or numbness of face or limb on one side as a stroke warning sign [14]. Bray et al. indicated that the unprompted recall of two or more of the most common stroke warning signs was 20% and increased to 53% after public multimedia campaigns [30]. Similarly, a study conducted in Iceland revealed that 31% of participants could identify two or more stroke warning signs [31]. Despite 31% not being relatively high, it was still much higher than the percentage of people who correctly identified the warning signs in Category A of our study.

Interventions such as educational programs and distributing posters conveying symptoms of stroke have demonstrated their effectiveness in increasing awareness of stroke warning signs and the importance of immediately seeking medical treatment [3],[32]. Although public education on stroke has been well-established in developed countries, similar efforts have not been invested in developing countries, and public education on stroke remains urgent in developing countries. Awareness of stroke warning signs is surprisingly low, and the majority of people do not undertake any stroke preventive actions. However, we also discovered that stoke patients in our study had significantly higher scores for Category A warning signs, which was expected as those signs may have been experienced prior to hospital admission. Our results and other findings mentioned above indicate that the general population without stroke experience requires urgent health education on stroke as it would be irrational and unproductive to allow people to learn about stroke warning signs through actual stroke experience. However, health education in a developing country such as Burkina Faso may be more difficult to implement than implementation in developed countries. Developing countries need to realize the importance of NCDs before any significant resource allocation towards eliminating these diseases can take place.

Less than half of the participants from the stroke group were unaware that they were at risk of stroke before stroke onset. Such beliefs hinder efforts for stroke prevention. Up to 15% of our respondents believed that strokes had supernatural causes. This result may indicate that they believe that stroke cannot be prevented through patient efforts. However, this may not be the case in developed countries. Thus, interventions that work for developed countries may have to be redesigned to suit the local conditions of developing countries, such as Burkina Faso. The extremely high percentage of respondents who undertook no stroke preventive actions and the high percentage of stroke patients who were unwilling to make efforts to reduce future stroke events emphasize the urgency for public education on stroke in Burkina Faso. However, such intervention may not be easy as it has been demonstrated that simple interventions such as repeated encouragement and verbal instruction had no effect on specific behavioral changes for stroke patients, even for developed countries [33].

## 5. Conclusions

Stroke is a major cause of death in Burkina Faso, and timely treatment is essential to reduce mortality. Knowledge and preventive efforts vary significantly between stroke patients and the general population. However, for both the general population and stroke patients, stroke knowledge and willingness to change behavior to prevent stroke or future recurrent stroke were extremely low. Public education on knowledge and strategies to improve willingness for stroke prevention remain urgent in African countries.
